# Behavioral phenotypes associated with cannabis and alcohol substitution

**DOI:** 10.1186/s12954-026-01432-y

**Published:** 2026-03-06

**Authors:** Claire L. Pince, Cianna J. Piercey, Vanessa T. Stallsmith, Katelyn Weldon, Jesse Ruehrmund, Gregory Dooley, Hollis C. Karoly

**Affiliations:** 1 Department of Psychology, Colorado State University, Fort Collins, CO USA; 2 Department of Psychology and Neuroscience, University of Colorado Boulder, Boulder, CO USA; 3 Department of Psychiatry, University of Colorado Anschutz Medical Campus, Aurora, CO USA; 4 Department of Environmental and Radiological Health Sciences, Colorado State University, CO Fort Collins, USA

**Keywords:** Alcohol, Cannabis, Substitution, Simultaneous use, Behavioral phenotyping

## Abstract

**Objective:**

Alcohol and cannabis co-use is the most common form of polysubstance use in the U.S. Some evidence suggests that there is an association between cannabis use and reduced alcohol consumption, however data on this topic are mixed. Further research is needed to elucidate the individual factors that determine whether cannabis substitutes for alcohol.

**Method:**

This study is an exploratory analysis of data from a within-subjects, crossover laboratory study on alcohol and cannabis co-administration. Participants completed two sessions: one in which they self-administered alcohol after using cannabis, and one in which they self-administered alcohol but did not use cannabis. We aim to compare behavioral phenotypes of individuals who self-administered less alcohol after cannabis (“substituters”; *n* = 23), more alcohol after cannabis (“complementers”; *n* = 7), or the same amount of alcohol regardless of whether cannabis was used (“non-substituters”; *n* = 16). We utilized Welch’s ANOVAs and Kruskal-Wallis tests to compare baseline group differences, drawing on domains from the Addictions Neuroclinical Assessment (ANA) framework. Wilcoxon tests were employed to test differences in laboratory self-administration measures between sessions for each group.

**Results:**

Substituters had significantly lower scores on measures corresponding to ANA domains of negative emotionality (BDI-II, DASS-Depression) and executive function (UPPS-P Lack of Premeditation) than non-substituters. Substituters endorsed higher positive cannabis expectancies than non-substituters, though expectancy differences did not reach significance.

**Conclusions:**

Individuals who use cannabis and alcohol and have lower levels of negative emotionality and impulsivity may be more likely to substitute cannabis for alcohol. Future research is needed to explore long-term outcomes of cannabis substitution.

**Clinical trials registration number:**

NCT04998006.

**Supplementary Information:**

The online version contains supplementary material available at 10.1186/s12954-026-01432-y.

## Introduction

Widespread legalization of cannabis in recent years has contributed to a steady increase in co-use of cannabis and alcohol in the U.S. [16], with recent estimates of 8.4% of adults aged 30–80 reporting current co-use [58]. Co-use of alcohol and cannabis includes use that occurs simultaneously (such that intoxicating effects overlap), as well non-overlapping (concurrent) use [55]. Co-use (particularly simultaneous co-use) has been associated with increased risk of poorer mental health and academic outcomes, engagement in risky behavior, and other harmful experiences, especially among young people [8, 28, 39, 47, 56]. Individuals often engage in simultaneous use for the “complementary” effects of the substances [7], which may lead to increased alcohol consumption during simultaneous use relative to alcohol-only use events [39, 55]. Conversely, there is also considerable evidence suggesting that for some individuals, using cannabis and alcohol at the same time, particularly when cannabis is used first, results in decreased alcohol consumption (i.e., “substitution”) and reduced craving [34, 41]. Further research is needed to elucidate individual factors associated with different patterns of alcohol and cannabis co-use.

The Addictions Neuroclinical Assessment (ANA) framework may offer insight into behavioral phenotypes that may be associated with specific alcohol-cannabis co-use patterns. The ANA framework has been proposed as a tool for developing precision medicine approaches to treating alcohol and substance use disorders (ASUDs), consisting of 3 functional domains that map onto the 3 stages of the addiction cycle [25, 27]. The Incentive Salience domain encapsulates processes such as attentional bias and cue-induced craving in response to drug-related stimuli, corresponding with the Binge-Intoxication stage of the addiction cycle [25, 27]. The Negative Emotionality domain reflects negative emotional states associated with addictive disorders, including withdrawal, reflecting the Withdrawal-Negative affect stage [25, 27]. Lastly, the Executive Function domain reflects dysfunction in processes related to planning and organizing goal-directed behaviors, which involves attention inhibition, working memory, and behavioral and cognitive adaptation, which maps onto the Preoccupation-Anticipation stage [27]. This framework allows deep phenotyping across these theory-derived functional domains, with the goal of identifying subgroups within a population who are most likely to respond to a given intervention [27]. While this framework was designed for use in clinical populations, a prior study validated a 4-factor structure in a sample of “problem drinkers” with and without AUD diagnoses, comprised of the three neurofunctional domains and an additional factor representing alcohol-related problems [36]. We propose that this framework may therefore offer utility in identifying behavioral phenotypes associated with cannabis and alcohol co-use patterns (e.g., substitution) in at-risk populations who do not necessarily have clinical diagnoses.

For example, a recent laboratory study employed a controlled-dose, parallel design to investigate the effect of smoked cannabis (< 0.3% placebo vs. 3.1%, 7.2% active THC) on alcohol consumption and craving in individuals who engage in heavy episodic drinking [34]. The 7.2% THC dose was associated with a significant reduction in alcohol urge after smoking (ANA incentive salience domain) compared to placebo and both active doses were associated with a significant reduction in alcohol consumed in an alcohol self-administration task compared to placebo [34]. This is consistent with findings from our crossover laboratory study wherein a subset of individuals experienced reductions in alcohol craving and consumption when cannabis was self-administered *ad libitum* before alcohol compared to when alcohol was self-administered without cannabis [41].

Several studies have also found associations between cannabis and alcohol co-use (both concurrent and simultaneous) and dimensions relevant to executive functioning, namely impulsivity [11, 43, 64, 66, 67] and challenges with emotion regulation [30, 35]. One study found that lower levels of positive urgency and lack of perseverance were associated with reduced alcohol consumption on co-use days relative to alcohol-only days (consistent with substitution) in a sample of veterans [66]. Daros et al. [11] also found there was a stronger positive relationship between grams of cannabis used and number of standard drinks consumed on a given day for young adults high in negative urgency and behavioral disinhibition [11]. Higher levels of impulsivity may therefore increase risk of alcohol-related harms in the context of simultaneous use. However, the relationship between grams of cannabis and any same day drinking was higher for individuals low in negative urgency and disinhibition, which the authors speculated may reflect planned, context-specific substance use (e.g., weekend social events) [11].

In terms of the ANA negative emotionality domain, prior studies have found associations between anxiety and depression symptoms and increased alcohol consequences and odds of AUD diagnosis for both concurrent and simultaneous alcohol and cannabis use [15, 33, 62, 63]. This suggests that greater negative emotionality may be a risk factor for greater alcohol related harms. Moreover, in an EMA study, Wycoff and Trull, [72] found that higher coping motives for drinking were associated with increases in anxious and depressed mood from the last moment, but this association was attenuated in simultaneous use relative to alcohol-only use moments [72]. This study did not explicitly show that lower negative emotionality is linked to substitution, but we hypothesize that the effect of simultaneous cannabis use on coping-motivated drinking could facilitate increased alcohol consumption in individuals with higher levels of negative affect via negative reinforcement mechanisms, based on these prior data. No prior study to date has explicitly examined how ANA phenotypes relate to substitution behavior during simultaneous use events in the laboratory.

To fill gaps in the literature and build upon prior data, the present secondary analysis is focused on linking ANA-derived behavioral phenotypes to alcohol and cannabis self-administration behavior in the lab. We leverage data from our aforementioned crossover laboratory study [41], involving two lab sessions: one “alcohol only” self-administration session and one session in which participants self-administered cannabis prior to alcohol self-administration (NCT04998006). Three groups (“substituters” who drank less during the Cannabis+Alcohol [CA] lab session compared to the Alcohol only [AO] lab session, “non-substituters”, who drank the same amount at both sessions, and “complementers”, who drank more at the CA session than the AO session) were derived from our data *post hoc*, based on observed drinking behavior during the CA and AO lab sessions [41]. Given emerging evidence from the ANA literature linking negative emotionality [15, 33, 62, 63, 72], impulsivity [60, 64, 66–68] and incentive salience [34, 65] to alcohol and cannabis co-use behavior, we hypothesized that substituters would have significantly lower scores across measures of trait impulsivity, negative emotionality, and incentive salience administered at baseline, compared to non-substituters and complementers.

## Methods

All methods were approved by Colorado State University’s Institutional Review Board. Data were collected as part of a larger study [41].

### Participants

Participants were recruited from the community using flyers, outreach at community events, and online advertisements. They met NIAAA heavy-drinking criteria (> 4 drinks on any day or > 14 drinks per week for males; > 3 drinks on any day or > 7 drinks per week for women) [38], endorsed using flower cannabis > = 3 times/week for at least three months, had not been diagnosed with an alcohol or substance use disorder (AUD/SUD) and were not currently seeking treatment. Exclusion criteria are described in the Supplementary Materials.

### Procedure

Eligible participants completed three appointments: one baseline session conducted in our on-campus lab and two sessions in our mobile laboratory, which travels to the participant’s residence and parks outside their home for the duration of the visit (Alcohol only session = AO, Cannabis + Alcohol session = CA). The mobile laboratory methodology allows for the study of legal market cannabis self-administration while complying with state and federal laws that prohibit researchers from bringing legal-market cannabis onto a university campus. Study appointments were separated by 2-weeks and the order of mobile laboratory visits was counterbalanced across participants.

### Baseline appointment

At the baseline appointment, participants underwent informed consent and eligibility verification procedures, including drug urinalysis (and pregnancy test when applicable) and a breathalyzer test. Eligible participants completed a series of surveys capturing demographics, substance use and psychological functioning, and medical and mental health history. Participants also completed an online timeline follow back documenting their substance use over the past 60 days (OTLFB; [32]). Participants were then provided with instructions for their mobile laboratory appointments. For the purposes of a separate study aim, participants were asked to try to abstain from cannabis use but to drink alcohol as they normally would up in the 2 weeks prior to session AO, up until 24 h before the mobile lab session. In the two weeks preceding session CA, participants were asked to use alcohol and cannabis as they normally would up until 24 h before the mobile lab session. Participants were asked not to use any drugs (including cannabis) or alcohol in the 24 h leading up to each mobile lab sessions.

### Mobile laboratory sessions

At both laboratory visits, participants completed a blood draw and were then given an alcohol priming drink (prepared using the Widmark formula targeting breath alcohol concentration [BrAC] of 0.03 g/dL). Next, (for both AO and CA) participants completed an alcohol self-administration task, wherein they had the opportunity to receive 4 drinks of their preferred alcohol (e.g. beer, wine, liquor) over the course of one hour (1 drink was offered every 15 minutes, with each drink targeting a 0.015 g/dL increase in BrAC), or alternatively receive an additional $1 per drink refused^1^, consistent with typical bar-lab procedures which offer small monetary incentives for not consuming drinks, to create a decisional balance between drinking and abstaining [45, 46]. Participants also completed questionnaires assessing alcohol craving throughout the self-administration period, for which the results are presented in Pince et al. [41]. All procedures were identical at sessions AO and CA up until the first blood draw, after which participants self-administered their own legal-market flower cannabis product inside their residence prior to receiving the priming drink.

There were three venous blood draws to assess levels of blood cannabinoids over the course of the study; one blood draw occurred before the priming drink at session AO and two occurred at session CA (one before cannabis self-administration and one immediately upon returning to the mobile lab after self-administering cannabis). The second blood draw post-cannabis use at session CA is used to verify that participants used cannabis during the session. Plasma was extracted from blood samples and stored at −80 °C until being assayed for THC and metabolites (11-OH-THC, THC-COOH) using High Performance Liquid Chromatography-Tandem Mass Spectrometry [9, 12, 23]. A detailed description of blood cannabinoid assay methods are included in Supplementary Materials. For further information on study procedures, see NCT04998006 and our prior publication [41].

### Measures

At the baseline visit, participants completed a series of surveys related to demographic characteristics, substance use, personality, and psychological functioning, which mapped onto ANA domains. Consistent with previous studies [18, 36, 37], we utilized the Penn Alcohol Craving Scale (PACS) [14] and the Alcohol Use Disorders Identification Test (AUDIT) [44] to capture the incentive salience domain. As the majority of research on ANA has focused on alcohol, we use comparable measures to capture cannabis-related incentive salience, including The Marijuana Dependence Scale (MDS) [52] and Marijuana Craving Questionnaire (MCQ) [22]. The AUDIT and MDS were also employed to screen for AUD and CUD symptomatology. For the negative emotionality domain, we used The Beck Depression Inventory II (BDI-II) [5] and Beck Anxiety Inventory II (BAI) [4], which have been employed in prior studies [27, 61]. We also included the Depression-Anxiety-Stress-Scale (DASS) to capture a wider range of negative affective states [29]. For the executive function domain, we used the Urgency, Premeditation, Perseverance, Sensation Seeking, and Positive urgency Impulsive Behavior Scale (UPPS-P) [10, 70], the Impaired Control Scale (ICS) [21 ], and Barratt Impulsiveness Scale (BIS-15) short-form [40, 48], which have been represented in prior ANA literature [18, 26, 36, 37, 50]. We also included the Impulsive Sensation Seeking Scale (ImpSS) [73], as impulsivity and sensation seeking personality traits represent potential risk and maintaining factors for SUDs relevant to the executive function ANA domain [57]. The ImpSS has been shown to predict substance use amongst undergraduates as well as relapse amongst individuals with SUDs [1, 20]. We also included the Difficulties in Emotion Regulation Scale (DERS) [6, 17], as emotion regulation engages cognitive control dimensions of executive functioning, which are thought to be disrupted in individuals with SUDs [31, 71]. A recent meta-analysis revealed that individuals with SUDs lower on the DERS, specifically in the strategies and impulse subscales [51]. A detailed description of each measure is included in the Supplementary Materials.

### Data analysis

Participants who completed both mobile laboratory sessions were categorized as either “substituters” (self-administered fewer drinks after using cannabis compared to alcohol alone), “complementers” (self-administered more drinks after using cannabis compared to alcohol alone), or “non-substituters” (self-administered the same number of drinks after using cannabis + alcohol compared to alcohol alone), as described in our prior publication [41]. Individuals who did not self-administer any alcohol during either laboratory session were excluded from the present analyses. We assessed group differences in baseline substance use, mobile laboratory measures, and measures of personality and psychological functioning, using Welch’s ANOVAs and Kruskal-Wallis non-parametric tests depending on variable distributions. Group differences in demographics were also evaluated using Fisher’s exact tests. When significant group differences were observed, post-hoc pairwise comparisons were conducted with a Bonferroni correction. Within-subject differences for mobile laboratory measures (e.g., blood THC, number of drinks self-administered) for each group were evaluated using pairwise Wilcoxon signed-rank tests. When statistically significant group differences in behavioral phenotyping measures were found, data were visualized with boxplots (non-significant statistics are reported in Supplementary Materials). Descriptive statistics for behavioral phenotyping measures for each group are included in the Supplementary Materials. All statistical analyses were performed using RStudio statistical software [42].

## Results

### Group characteristics

Sixty-one participants completed both mobile laboratory appointments. Participants who chose not to self-administer any alcohol at either appointment (*n* = 15) were excluded from analyses, resulting in a final sample of 46, 50% of which were classified as substituters (self-administered at least one more drink at session AO compared to CA; *n* = 23), 34.7% as non-substituters (self-administered the same number of drinks at session AO and CA; *n* = 16), and 15.3% as complementers (self-administered at least one more drink at session CA compared to AO; *n* = 7). Based on clinical cutoffs from the Alcohol Use Disorders Identification Test and Marijuana Dependence Scale, a majority of our final sample (64.7%) met criteria for at least mild AUD (AUDIT > = 8) and almost half (43.5%) met criteria for CUD (MDS > = 3) at baseline, nearly a third of which met criteria for both AUD and CUD (28.2%). Only 8 individuals (17.4%) didn’t meet criteria for either diagnosis based on AUDIT and MDS scores.

Regarding baseline substance use, Kruskal-Wallis tests revealed significant group differences in typical drinks per drinking day in the past 2 months (*χ*^2^(2) = 7.63, *p* <.05). There were trends towards non-substituters consuming more drinks per drinking day compared to complementers (*Z* = 2.36, *p* =.06) and substituters (*Z* = 2.31, *p* =.06). There were no other significant group differences in baseline alcohol use frequency or cannabis use frequency and quantity, nor did Fisher’s tests reflect significant demographic differences between groups (Table 1). In the mobile laboratory, substituters self-administered an average of 2.96 (*SD* = 1.19) drinks at session AO and 1 drink (*SD* = 1.21) at session CA, with a Wilcoxon test revealing a statistically significant difference (*Z* = −4.18, *p* <.001) with a large effect size (*r* =.87). Non-substituters self-administered an average of 3.19 drinks (*SD* = 1.22) at both sessions. Complementers self-administered an average of 0.71 drinks (*SD* = 0.76) at session AO and 2.71 drinks (*SD* = 1.38) at session CA, which was a statistically significant difference (*Z* = −2.28, *p* <.001) with a large effect size (*r* =.86). Kruskal-Wallis tests revealed significant group differences in drinks self-administered at session AO (*χ*^2^(2) = 14.61, *p* <.001) and session CA (*χ*^2^(2) = 20.55, *p* <.001). Complementers self-administered significantly fewer drinks at session AO compared to non-substituters (*Z* = −3.67, *p* <.001) and substituters (*Z* = −3.41, *p* =.001), but there were no significant differences between non-substituters and substituters (*Z* = 0.60, *p* =.55). In contrast, substituters self-administered significantly fewer drinks at session CA compared to non-substituters (*Z* = −4.44, *p* <.001) and complementers (*Z* = −2.59, *p* =.02) (Table 2).

**Table 1:** Group demographics and baseline substance use

	Substituter (*n* = 23)	Non-substituter (*n* = 16)	Complementer (*n* = 7)
*Demographics*			
Gender, n (%)			
Cisgender Man	16 (69.6%)	9 (56.25%)	2 (28.6%)
Cisgender Woman	6 (26.1%)	7 (43.75%)	4 (57.1%)
Non-binary	1 (4.3%)	0 (0%)	1 (14.3%)
Sex assigned at birth, Male, n (%)	16 (69.6%)	9 (56.3%)	3 (42.8%)
Age, mean (S.D.)	31.9 (11.2)	30.3 (9.0)	27.6 (7.0)
Race/Ethnicity, n (%)			
Asian	1 (4.3%)	0 (0%)	0 (0%)
Black or African American	1 (4.3%)	0 (0%)	0 (0%)
Hispanic/Latino	4 (17.4%)	0 (0%)	1 (14.3%)
Multiracial/ethnic	3 (13%)	0 (0%)	0 (0%)
White	14 (61%)	16 (100%)	6 (85.7%)
Education, n (%)			
High School	1 (4.3%)	3 (18.75%)	0 (0%)
Some college	5 (21.8%)	4 (25.0%)	4 (57.1%)
Associates/Technical degree	5 (21.8%)	2 (12.5%)	0 (0%)
Bachelor’s degree	10 (43.5%)	5 (31.25%)	3 (42.8%)
Master’s degree	1 (4.3%)	2 (12.5%)	0 (0%)
Doctoral degree	1 (4.3%)	0 (0%)	0 (0%)
Employment, n (%)			
Full-time	9 (39.1%)	7 (43.75%)	3 (42.8%)
Part-time	6 (26.1%)	3 (18.75%)	1 (14.3%)
Unemployed, disabled, or retired	4 (17.4%)	2 (12.5%)	1 (14.3%)
Student full-time	3 (13%)	3 (18.75%)	2 (28.6%)
Stay-at-home parent	1 (4.4%)	1 (6.25%)	0 (0%)
Annual Income, n (%)			
Less than $10,000	5 (21.75%)	5 (31.25%)	1 (14.3%)
$10,000–20,000	2 (8.7%)	1 (6.25%)	0 (0%)
$20,000–30,000	5 (21.75%)	4 (25.0%)	1 (14.3%)
$30,000–40,000	2 (8.7%)	0 (0%)	1 (14.3%)
$40,000–50,000	1 (6.25%)	1 (6.25%)	1 (14.3%)
$50,000–60,000	6 (26.1%)	3 (18.75%)	3 (42.8%)
Greater than $60,000	2 (6.25%)	1 (6.25%)	0 (0%)
*Baseline substance use characteristics*			
Alcohol use days in past 60 days (O-TLFB), mean (S.D.)	21.70 (17.20)	25.62 (15.18)	31.43 (14.52)
Drinks per drinking day (O- TLFB), mean (S.D.)^1^	3.60 (1.27) ^1^	5.20 (2.02) ^1^	3.41 (1.91)
Flower cannabis days in past 60 days (O-TLFB), mean (S.D.) ^1^	31.3 (22.3)^1^	31.44 (21.39)	25.14 (24.60)
Grams flower cannabis used per day (O-TLFB), mean (S.D.) ^1^	0.65 (0.70)	0.53 (0.47)	0.27 (0.10)

^1^data missing for one participant; ^2^data missing from two participants

**Table 2:** Group mobile laboratory self-administration descriptives

	Substituter (*n* = 23)	Non-substituter (*n* = 16)		Complementer (*n* = 7)
*Alcohol self-administration*				
Drinks self-administered				
Session AO, mean (S.D.)	2.96 (1.19)	3.19 (1.22)^###^		0.71 (0.76)^###^
Session CA, mean (S.D.)	1.00 (1.21)	3.19 (1.22)^###^		2.71 (1.38)^#^
Session CA vs. AO, mean difference	1.96 (1.11)***	0 (0.00)		−2.00 (1.29)**
*Cannabis self-administration*				
Grams flower cannabis, mean (S.D.)	0.46 (0.61)	0.42 (0.58)		0.25 (0.17)
THC percentage, mean (S.D.)	23.2 (5.05)	23.1 (4.71)		20.1 (2.98)
Flower cannabis strain. n (%)^1^				
Sativa	9 (45%)	5 (31.25%)		2 (28.6%)
Indica	4 (25%)	2 (12.5%)		3 (42.8%)
Hybrid	7 (35%)	9 (56.25%)		2 (28.6%)
*Blood cannabinoid concentrations (ng/mL)* ^*2*^				
Blood THC concentration, mean (S.D.)				
Baseline AO	9.65 (19.0)	5.78 (7.99)		12.01 (15.45)
Baseline CA	11.72 (23.03)	4.34 (5.16)		7.30 (14.16)
Post-use CA	193.26 (229.16)	140.39 (159.70)		129.84 (135.25)
Baseline CA vs. AO difference	2.47 (6.63)*	−0.90 (3.75)		0.15 (11.31)
Baseline CA vs. Post-use difference	190.59 (216.61)***	126.20 (157.09)***		117.68 (123.52)*
Blood 11-OH-THC concentration, mean (S.D.)				
Baseline AO	3.43 (5.16)	2.42 (3.58)		4.94 (7.15)
Baseline CA	3.62 (4.95)	1.87 (2.25)		6.41 (7.30)
Post-use CA	11.08 (11.09)	16.01 (17.67)		20.07 (21.50)
Baseline CA vs. AO difference	0.52 (1.52)	−0.38 (1.55)		1.47 (6.31)
Baseline CA vs. Post-use difference	7.58 (8.47)***	12.62 (15.72)***		13.65 (15.49)*
Blood THC-COOH concentration, mean (S.D.)				
Baseline AO	78.23 (111.29)	69.03 (102.62)		96.98 (153.07)
Baseline CA	86.88 (115.61)	65.74 (104.87)		86.76 (101.12)
Post-use CA	128.81 (138.04)	135.75 (166.44)		119.67 (130.49)
Baseline CA vs. AO difference	17.30 (45.70)	2.24 (35.04)		−10.23 (60.71)
Baseline CA vs. Post-use difference	39.19 (73.36)**	54.18 (58.38)***		32.92 (31.52)*

^1^*n* = 43, missing strain information for 3 substituters; ^2^7 individuals had one missing blood timepoint, 2 had two missing timepoints and 3 had three, due to difficulty with blood draws. ^#^*p* <.05, ^###^ *p* <.001, between-group, within-timepoint comparison; *p <.05, ** *p* <.01, ***p <.001, within-group, between-timepoint comparison

Regarding cannabis self-administration, Kruskal-Wallis tests indicated no significant group differences in the amount or potency of flower cannabis used during session CA. On average, participants self-administered an average of 0.37 g (*SD* = 0.48) of flower cannabis with a THC content of 22.82% (*SD* = 22.48). All groups experienced a significant increase in blood THC concentrations between baseline and post-cannabis use (*p* <.05). On average, baseline blood THC concentration was 8.61 ng/mL (*SD* = 15.15) at session AO and 9.02 ng/mL (*SD* = 17.13) at CA, with an average post-use concentration of 165.24 ng/mL (*SD* = 193.00). While there were no significant group differences in blood THC or metabolites at any timepoint, Wilcoxon tests revealed that only substituters had significantly higher baseline blood THC concentrations at session CA compared to AO (*Z* = 2.25, *p* =.02), with a medium effect size (*r* =.47) (Table 2).

### Incentive salience measures

**Fig. 1:**
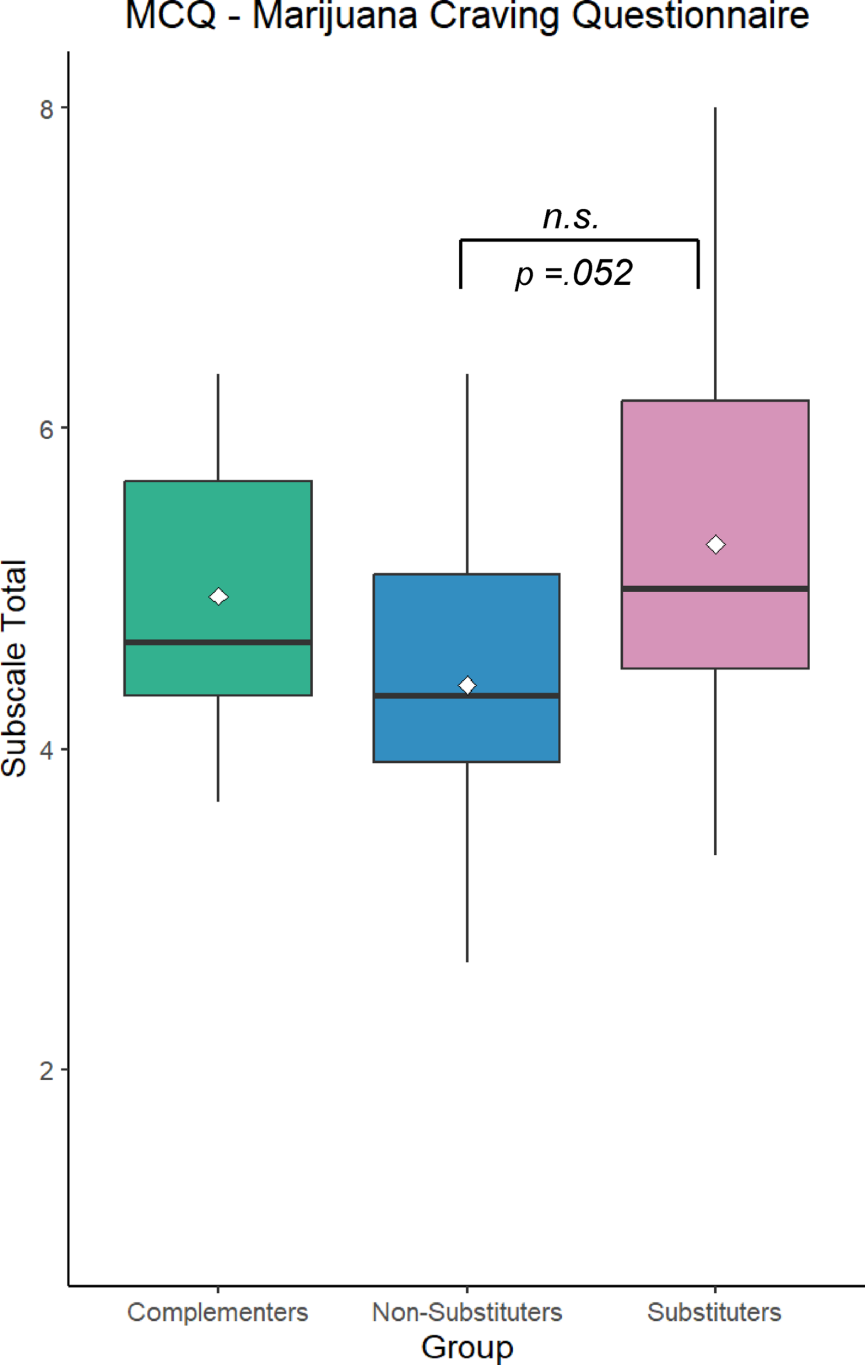
Substituters trended towards having significantly higher scores on the Marijuana Craving Questionnaire Expectancy subscale compared to Non-substituters

A Welch’s ANOVA revealed a trend towards a significant group difference for the Expectancy subscale of the MCQ (*F*_*w*_(17.8,2) = 2.87, *p* =.08), with post-hoc analyses indicating a trend towards substituters scoring higher than non-substituters (*p* =.052) (Fig. 1). There were no significant group differences for any other subscales of the MCQ nor the PACS, MDS, or AUDIT (Supplementary Materials).

### Negative emotionality measures

**Fig. 2:**
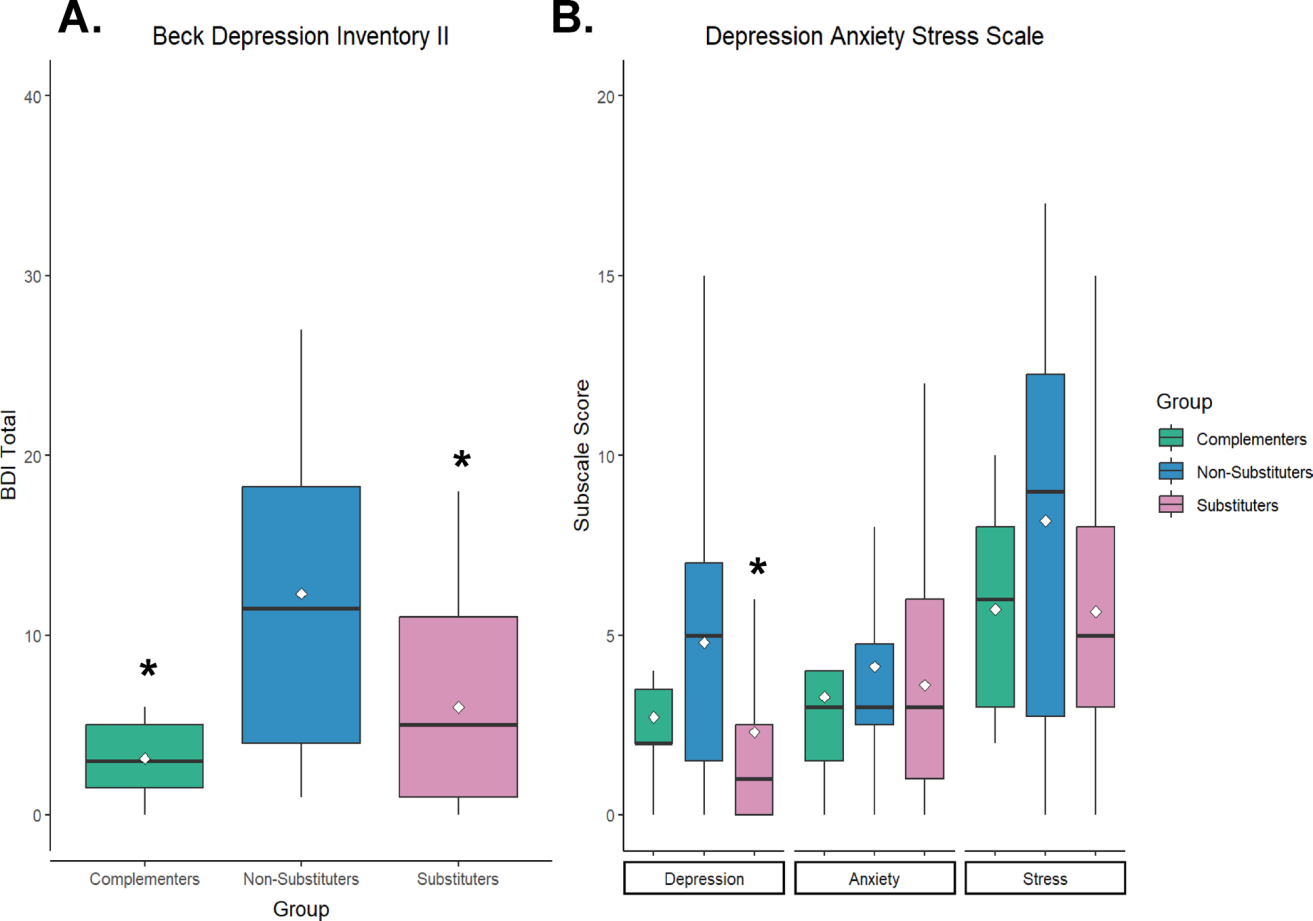
**A** Substituters and complementers had significantly lower scores on the Beck Depression Inventory II (BDI-II) compared to non-substuters. **B** Substituters scored significantly lower on the Depression subscale of the Depression Anxiety Stress Scale (DASS) compared to non-substituters. *p < .05

A Kruskal-Wallis test indicated significant group differences in scores on the BDI-II (*χ*^2^(2) = 8.79, *p* =.01), with a large effect size (*η*^2^ = 0.16). Post-hoc analyses revealing that substituters (*Z* = −2.44, *p* =.04) and complementers (*Z* = −2.55, *p* =.03) scored significantly lower than non-substituters. There were also significant group differences in DASS Depression subscale scores (*χ*^2^(2) = 6.60, *p* =.04), with a medium effect size (*η*^2^ = 0.11). Post-hoc analyses indicated substituters scored significantly lower than non-substituters (*Z* = −2.56, *p* =.03) (Fig. 2). There were no significant group differences in BAI scores or DASS Stress or Anxiety subscales (Supplementary Materials).

### Executive function measures

**Fig. 3:**
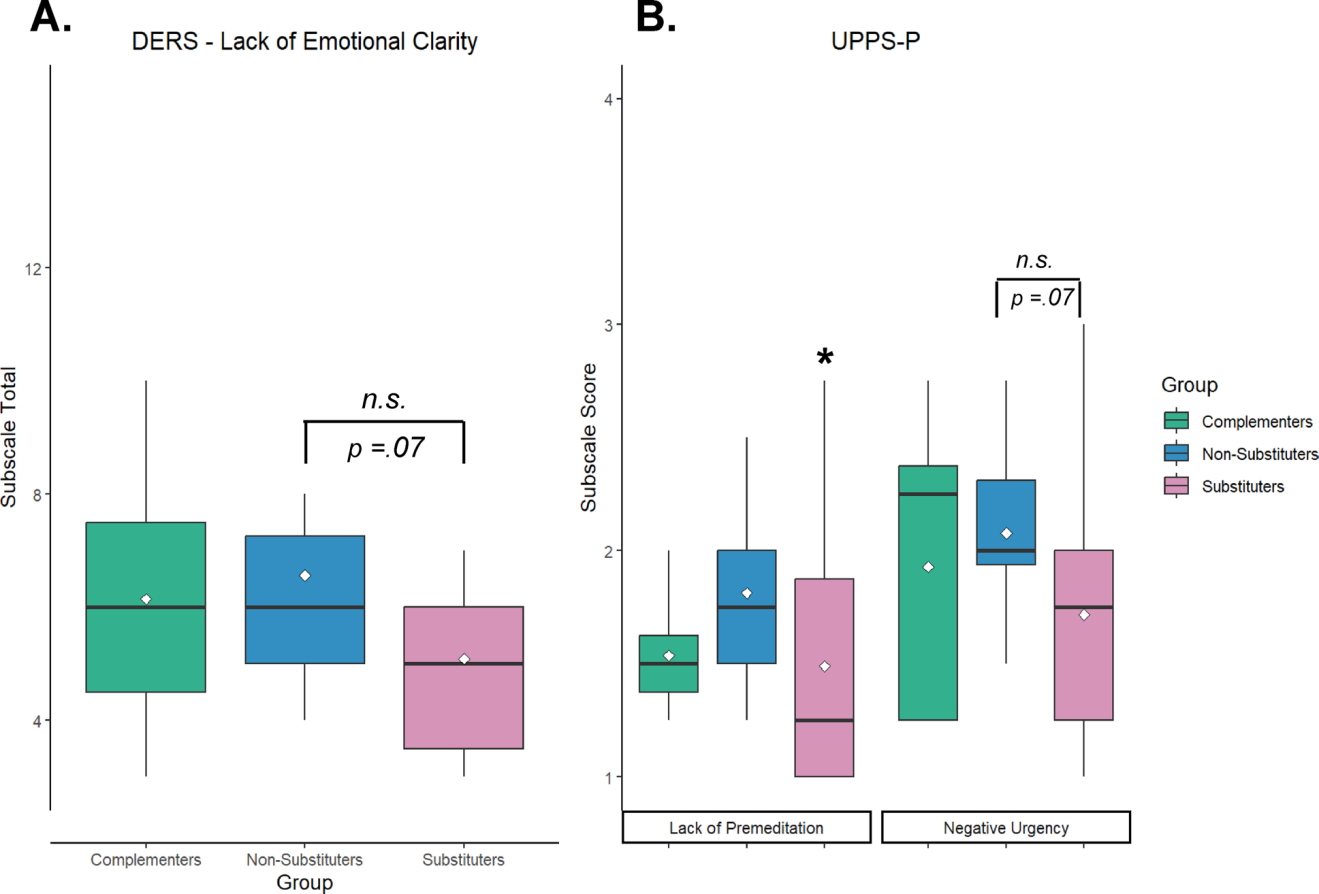
**A** Substituters trended towards lower scores on the Difficulties in Emotion Regulation Scale (DERS) Lack of Emotional Clarity subscale compared to non-substituters (*p* = .07). **B** Substituters scored significantly lower on the Lack of Premeditation subscale of the Urgency, Premeditation, Perseverance, Sensation Seeking, and Positive urgency Impulsive Behavior Scale (UPPS-P) compared to non-substituters. *p < .05

A Kruskal-Wallis test revealed significant group differences in UPPS-P Lack of Premeditation subscale scores (*χ*^2^(2) = 6.06, *p* = .048), with a medium effect size (*η*^2^ = 0.09). Post-hoc analyses showed that substituters scored significantly lower than non-substituters (*Z* = −2.45, *p* =.04). There was also a trend towards group differences in UPPS-P Negative Urgency subscale scores (*χ*^2^(2) = 5.32, *p* =.07), with post-hoc tests indicating substituters scored lower than non-substituters (*Z* = −2.25, *p* =.07). There were no significant differences between complementers and substituters or non-substituters on either of these subscales. A Kruskal-Wallis test demonstrated a trend towards a difference in the Lack of Emotional Clarity subscale of the DERS (*χ*^2^(2) = 5.55, *p* =.06), whereby substituters scored lower than non-substituters (*Z* = 2.26, *p* =.07) (Fig. 3). There were no significant group differences in scores on any other DERS subscales, nor ImpSS, ICS, or BIS subscales (Supplementary Materials).

## Discussion

In this novel laboratory study, we used the ANA framework [27] to characterize behavioral phenotypes associated with alcohol and cannabis self-administration behavior, as no studies to date have examined how ANA phenotypes relate to co-use behavior during simultaneous use events in the laboratory. We hypothesized that substituters would have significantly lower scores across measures of impulsivity, negative emotionality, and incentive salience administered at baseline, compared to non-substituters and complementers. Findings partially supported hypotheses, as both substituters and complementers reported significantly lower levels of depression compared to non-substituters. Substituters also endorsed significantly lower lack of premeditation than non-substituters.

Higher scores on lack of premeditation and depression among non-substituters in the present study may reflect a behavioral pattern of impulsive drinking during co-use to alleviate negative affect [2, 49, 59, 66, 72], whereas lower depression and greater premeditation among substituters may contribute to more conscientious drinking choices [11]. In recent work, the simultaneous use of cannabis with alcohol has been shown to attenuate increases in negative affect associated with coping-motivated drinking, which may reflect an underlying negative reinforcement mechanism unique to simultaneous use [72]. Further, recent work by Wardell and colleagues [69] showed a within-person association between internalizing symptoms (i.e., depression, anxiety, and stress) and number of heavy drinking simultaneous use days via reward/enhancement motives [69]. However, at the between-person level, greater average internalizing symptoms were associated with more light drinking simultaneous use days and fewer heavy drinking simultaneous use days via coping motives [69], highlighting the importance of distinguishing between use motives, trait-level emotional vulnerability vs. state-dependent use patterns, and potential behavioral differences across substance use contexts (i.e., self-report vs. laboratory). It is notable that both substituters and complementers demonstrated significantly lower levels of depression than non-substituters in the present analysis. While lower negative emotionality among complementers compared to non-substituters may reflect drinking motives aimed at enhancing positive mood during simultaneous use events [54], we are hesitant to draw conclusions about this finding give the small (*n* = 7) subset of participants in the complementers group.

Prior studies have found associations between trait impulsivity and quantity of alcohol consumed on co-use days [11, 66], though our study was the first to investigate the association between trait impulsivity and substitution in the laboratory. We did not observe significant group differences in positive urgency and lack of perseverance, which have been shown to predict alcohol consumption on cannabis use days (i.e., substitution) among veterans [66]. Instead, we found that substituters had lower lack of premeditation and a trend towards lower negative urgency. Consistent with our findings, negative urgency has previously been shown to moderate the association between grams of cannabis used and number of drinks consumed in a given day for young adults, with higher negative urgency corresponding to greater quantities of cannabis and alcohol use [11]. However, Rothstein and Stamates, [43] found that lack of premeditation was associated with lower co-use frequency in college students [43]. There is also evidence to suggest planned simultaneous use is associated with greater alcohol consumption and intoxication [13, 53], emphasizing a need for further research on the role of premeditation in co-use behavior in naturalistic settings.

Within the ANA incentive salience domain, no significant group differences emerged, potentially reflecting homogeneity introduced by study inclusion criteria related to alcohol and cannabis use characteristics (i.e., participants were not recruited on the basis of being a clinical sample and had relatively low levels of craving and dependence symptoms for both alcohol and cannabis) [41]. Thus, incentive salience may be less relevant than other ANA domains for this specific population. Overall, findings support the ANA’s emphasis on negative emotionality and executive function as core domains in addiction phenotyping, underscoring the importance of individual differences when considering cannabis as a potential substitute for alcohol. In addition, our study extends prior work by applying the ANA framework in a non-clinical sample of individuals engaged in alcohol and cannabis co-use in the laboratory and provides further support for the potential utility of the ANA in non-clinical samples [36].

### Limitations and future directions

Demographic diversity within our sample was limited, and we did not explicitly recruit a clinical sample for this study. However, many of these individuals are likely to meet criteria for AUD and CUD based on their scores on baseline screening measures. Replication in a clinical sample with greater diversity is necessary to extend the generalizability of findings. We were also limited in our ability to draw conclusions regarding the complementer group due to the small sample size. Due to federal restrictions, participants consumed commercial flower cannabis products *ad libitum* during the cannabis and alcohol co-administration session, thus we lacked experimental control over cannabis dosage, strain, and mode of administration. Future studies should employ stricter dosing controls and could also explore co-administration of alcohol alongside other forms of legal-market cannabis, such as cannabis concentrates [24]. We also only employed one order of co-administration (cannabis before alcohol), limiting our ability to investigate order effects. Participants were also asked to abstain from cannabis use prior to session AO and therefore may have self-administered more alcohol due to cannabis withdrawal. Additionally, while we used behavior during a single simultaneous use event in the laboratory to categorize participants, engagement in substitutive vs. complementary use of cannabis and alcohol may vary within person at the momentary level [19], which should be further examined using ecological momentary assessment (EMA) or other experience sampling methods in combination with laboratory assessments. Such a design would allow for the assessment of contextual factors and consequences associated with substitution and co-use. Future research may also benefit from assessing state-level affect (e.g., depression, anxiety) prior to drug administration and including a measure of co-use expectancies at baseline [3]. Finally, we acknowledge that our participants were only offered $1 per drink not consumed, which is slightly lower than the amount offered in recent bar-lab studies (e.g., $2 per drink) [45, 46]. This could have promoted greater alcohol consumption during the sessions. However, given that the same $1 incentive was used at both lab sessions, this does not meaningfully impact our interpretation of substitution effects in the present study.

## Conclusions

The present findings offer preliminary evidence that individual differences in negative emotionality and impulsivity may meaningfully shape alcohol and cannabis co-use behavior. Notably, substitution of cannabis for alcohol in the laboratory was associated with lower levels of negative emotionality and impulsivity. These results, while preliminary, underscore the potential utility of the ANA framework to identify individuals who may be more or less likely to acutely reduce their alcohol intake while using cannabis, with possible implications for tailoring interventions or harm-reduction strategies for people who co-use alcohol and cannabis. However, given that we did not link the present findings to real-world alcohol or cannabis related consequences, additional longitudinal research is needed to establish whether these results extend to real-world use patterns and consequences over time, and to determine whether substitution may be a reasonable harm-reduction strategy for some individuals who wish to reduce their alcohol use.

## Supplementary Information

Supplementary Material 1

## Data Availability

Data and materials are available from the corresponding authors upon reasonable request.

## Footnotes

1 Note that $5 per drink was given for the first 2 participants (at both sessions AO and CA). However, we ultimately decided based on participant feedback and input from members of the study team that $1 per drink was a more appropriate incentive, given the mobile laboratory setting is highly dissimilar from participants’ typical drinking environment and from previous bar-lab studies. We acknowledge that this amount is lower than the typical monetary incentive used in previous bar-lab paradigms and discuss this in the limitations section.

## References

[1] Abdelhamid EA, Hammad HA-H, Morsy OMI. The interplay of cognitive distortions, impulsive sensation seeking, and relapse probability among clients with substance use disorders. Tanta Sci Nurs J. 2024;34(3):0–0. 10.21608/tsnj.2024.375867.

[2] Adams ZW, Kaiser AJ, Lynam DR, Charnigo RJ, Milich R. Drinking motives as mediators of the impulsivity-substance use relation: pathways for negative urgency, lack of premeditation, and sensation seeking. Addict Behav. 2012;37(7):848–55. 10.1016/j.addbeh.2012.03.016. 22472524 10.1016/j.addbeh.2012.03.016 PMC3356454

[3] Barnwell SS, Earleywine M. Simultaneous alcohol and cannabis expectancies predict simultaneous use. Subst Abuse Treat Prev Policy. 2006;1(1):29. 10.1186/1747-597X-1-29. 17034634 10.1186/1747-597X-1-29 PMC1624811

[4] Beck AT, Epstein N, Brown G, Steer R. Beck anxiety inventory. 1988. 10.1037/t02025-000

[5] Beck AT, Steer RA, Brown G. Beck Depression Inventory–II. 1996. 10.1037/t00742-000

[6] Bjureberg J, Ljótsson B, Tull MT, Hedman E, Sahlin H, Lundh L-G, et al. Development and validation of a brief version of the difficulties in emotion regulation scale: the DERS-16. J Psychopathol Behav Assess. 2016;38(2):284–96. 10.1007/s10862-015-9514-x. 27239096 10.1007/s10862-015-9514-x PMC4882111

[7] Boyle HK, Gunn RL, López G, Fox OS, Merrill JE. Qualitative examination of simultaneous alcohol and cannabis use reasons, evaluations, and patterns among heavy drinking young adults. Psychol Addict Behav. 2021;35(6):638–49. 10.1037/adb0000746. 34472878 10.1037/adb0000746 PMC8487895

[8] Bravo AJ, Prince MA, Pilatti A, Mezquita L, Keough MT, Hogarth L. Young adult concurrent use and simultaneous use of alcohol and marijuana: a cross-national examination among college students in seven countries. Addict Behav Rep. 2021;14:100373. 10.1016/j.abrep.2021.100373. 34938834 10.1016/j.abrep.2021.100373 PMC8664774

[9] Conner BT, Smith E, DiCecco S, Sturgess K, Karoly HC, Dooley G, Akagi N, Villanueva C, Hennesy M. Pharmacokinetic differences between fast-acting, standard, and placebo cannabis edibles. (under review). 10.1177/25785125261441366 41958200

[10] Cyders MA, Littlefield AK, Coffey S, Karyadi KA. Examination of a short version of the UPPS-P impulsive behavior scale. Addict Behav. 2014;39(9):1372–6. 10.1016/j.addbeh.2014.02.013. 24636739 10.1016/j.addbeh.2014.02.013 PMC4055534

[11] Daros AR, Pereira BJ, Khan D, Ruocco AC, Quilty LC, Wardell JD. Daily associations between cannabis use and alcohol use in young adults: the moderating role of self-report and behavioral measures of impulsivity. Addict Res Theory. 2022;30(2):79–88. 10.1080/16066359.2021.1939314.

[12] Dooley G, Godbole S, Wrobel J, Henthorn TK, Brooks-Russell A, Limbacher S, Kosnett M. Comparison of ∆9-Tetrahydrocannabinol in venous and capillary blood following ad libitum cannabis smoking by occasional and daily users (under review). 10.1093/jat/bkaf043 PMC12716463 40366742

[13] Fairlie AM, Graupensperger S, Duckworth JC, Patrick ME, Lee CM. Unplanned versus planned simultaneous alcohol and marijuana use in relation to substance use and consequences: results from a longitudinal daily study. Psychol Addict Behav. 2021;35(6):712–22. 10.1037/adb0000738. 34591512 10.1037/adb0000738 PMC8484779

[14] Flannery BA, Roberts AJ, Cooney N, Swift RM, Anton RF, Rohsenow DJ. The role of craving in alcohol use, dependence, and treatment. Alcohol Clin Exp Res. 2001;25(2):299–308. 10.1111/j.1530-0277.2001.tb02213.x. 11236847

[15] Fleming CB, Duckworth JC, Rhew IC, Abdallah DA, Guttmannova K, Patrick ME, et al. Young adult simultaneous alcohol and marijuana use: between- and within-person associations with negative alcohol-related consequences, mental health, and general health across two-years. Addict Behav. 2021;123:107079. 10.1016/j.addbeh.2021.107079. 34403871 10.1016/j.addbeh.2021.107079 PMC8419075

[16] Gonçalves PD, Levy NS, Segura LE, Bruzelius E, Boustead AE, Hasin DS, et al. Cannabis recreational legalization and prevalence of simultaneous cannabis and alcohol use in the United States. J Gen Intern Med. 2023;38(6):1493–500. 10.1007/s11606-022-07948-w. 36451010 10.1007/s11606-022-07948-w PMC10160263

[17] Gratz KL, Roemer L. Multidimensional assessment of emotion regulation and dysregulation: development, factor structure, and initial validation of the difficulties in emotion regulation scale. J Psychopathol Behav Assess. 2004;26(1):41–54. 10.1023/B:JOBA.0000007455.08539.94.

[18] Gunawan T, Luk JW, Schwandt ML, Kwako LE, Vinson T, Horneffer Y, et al. Factors underlying the neurofunctional domains of the addictions neuroclinical assessment assessed by a standardized neurocognitive battery. Transl Psychiatry. 2024;14(1):271. 10.1038/s41398-024-02987-9. 38956031 10.1038/s41398-024-02987-9 PMC11219746

[19] Gunn RL, Aston ER, Metrik J. Patterns of cannabis and alcohol co-use: substitution versus complementary effects. Alcohol Res Curr Rev. 2022. 10.35946/arcr.v42.1.04. 10.35946/arcr.v42.1.04 PMC8855954 35223338

[20] Hamdan -MAM, Mahmoud KF, Al SAN, Arabiat DH. Impulsivity and sensation-seeking personality traits as predictors of substance use among university students. J Psychosoc Nurs Ment Health Serv. 2018;56(1):57–63. 10.3928/02793695-20170905-04. 28892553 10.3928/02793695-20170905-04

[21] Heather N, Tebbutt JS, Mattick RP, Zamir R. Development of a scale for measuring impaired control over alcohol consumption: a preliminary report. J Stud Alcohol. 1993;54(6):700–9. 10.15288/jsa.1993.54.700. 8271806 10.15288/jsa.1993.54.700

[22] Heishman SJ, Arasteh K, Stitzer ML. Comparative effects of alcohol and marijuana on mood, memory, and performance. Pharmacol Biochem Behav. 1997;58(1):93–101. 10.1016/S0091-3057(96)00456-X. 9264076 10.1016/s0091-3057(96)00456-x

[23] Henthorn TK, Wang GS, Dooley G, Brooks-Russell A, Wrobel J, Limbacher S, et al. Dose estimation utility in a population pharmacokinetic analysis of inhaled Δ9-tetrahydrocannabinol cannabis market products in occasional and daily users. Ther Drug Monit. 2024;46(5):672–80. 10.1097/FTD.0000000000001224. 39235358 10.1097/FTD.0000000000001224 PMC11389879

[24] Karoly HC, Prince MA, Emery NN, Smith EE, Piercey CJ, Conner BT. Protocol for a mobile laboratory study of co-administration of cannabis concentrates with a standard alcohol dose in humans. PLoS One. 2022;17(11):e0277123. 10.1371/journal.pone.0277123. 36327298 10.1371/journal.pone.0277123 PMC9632794

[25] Koob GF, Volkow ND. Neurocircuitry of addiction. Neuropsychopharmacology. 2010. 10.1038/npp.2009.110. 19710631 10.1038/npp.2009.110 PMC2805560

[26] Kwako LE, Momenan R, Grodin EN, Litten RZ, Koob GF, Goldman D. Addictions neuroclinical assessment: a reverse translational approach. Neuropharmacology. 2017;122:254–64. 10.1016/j.neuropharm.2017.03.006. 28283392 10.1016/j.neuropharm.2017.03.006 PMC5569299

[27] Kwako LE, Momenan R, Litten RZ, Koob GF, Goldman D. Addictions neuroclinical assessment: a neuroscience-based framework for addictive disorders. Biol Psychiatry. 2016;80(3):179–89. 10.1016/j.biopsych.2015.10.024. 26772405 10.1016/j.biopsych.2015.10.024 PMC4870153

[28] Looby A, Prince MA, Villarosa-Hurlocker MC, Conner BT, Schepis TS, Bravo AJ. Young adult use, dual use, and simultaneous use of alcohol and marijuana: an examination of differences across use status on marijuana use context, rates, and consequences. Psychol Addict Behav. 2021;35(6):682–90. 10.1037/adb0000742. 34591517 10.1037/adb0000742 PMC8484769

[29] Lovibond PF, Lovibond SH. The structure of negative emotional states: comparison of the depression anxiety stress scales (DASS) with the beck depression and anxiety inventories. Behav Res Ther. 1995;33(3):335–43. 10.1016/0005-7967(94)00075-u. 7726811 10.1016/0005-7967(94)00075-u

[30] Lucke HR, Harbke CR, Mathes EW, Hammersley JJ. Higher emotion dysregulation and coping motives in alcoholand marijuana users. Substance Use & Misuse. 2021;56(7):950–61. 10.1080/10826084.2021.1901927. 33754955 10.1080/10826084.2021.1901927

[31] Marceau EM, Kelly PJ, Solowij N. The relationship between executive functions and emotion regulation in females attending therapeutic community treatment for substance use disorder. Drug Alcohol Depend. 2018;182:58–66. 10.1016/j.drugalcdep.2017.10.008. 29154148 10.1016/j.drugalcdep.2017.10.008

[32] Martin-Willett R, Helmuth T, Abraha M, Bryan AD, Hitchcock L, Lee K, et al. Validation of a multisubstance online timeline followback assessment. Brain Behav. 2020;10(1):e01486. 10.1002/brb3.1486. 31793226 10.1002/brb3.1486 PMC6955818

[33] McCarty KN, Stevens AK, Gunn RL, Borsari B, Metrik J. Prospective associations between anxiety sensitivity, distress intolerance, depressive symptoms, and indices of alcohol and cannabis use among veterans. J Stud Alcohol Drugs. 2023;84(4):535–45. 10.15288/jsad.22-00257. 37096769 10.15288/jsad.22-00257 PMC10488313

[34] Metrik J, Aston ER, Gunn RL, Swift R, MacKillop J, Kahler CW. Acute effects of cannabis on alcohol craving and consumption: a randomized controlled crossover trial. Am J Psychiatry. 2025;0. 10.1176/appi.ajp.20250115. appi.ajp.20250115. 10.1176/appi.ajp.20250115 PMC12812287 41254853

[35] Moskal KR, Teeters JB, McCollum DC. Examining differences in emotion dysregulation between emerging adult alcohol-only users, abstainers, and simultaneous users. Cannabis. 2023;6(3):34–48. 10.26828/cannabis/2023/000166. 38035171 10.26828/cannabis/2023/000166 PMC10683745

[36] Nieto SJ, Grodin EN, Green R, Ray LA. Evaluation of the addictions neuroclinical assessment (ANA) framework through deep phenotyping of problem drinkers. Drug Alcohol Depend. 2021;221:108603. 10.1016/j.drugalcdep.2021.108603. 33618192 10.1016/j.drugalcdep.2021.108603 PMC8026564

[37] Nieto SJ, Grodin EN, Ray LA. Neural correlates of the addictions neuroclinical assessment (ANA) incentive salience factor among individuals with alcohol use disorder. Behav Brain Res. 2024;464:114926. 10.1016/j.bbr.2024.114926. 38431152 10.1016/j.bbr.2024.114926 PMC11563703

[38] NSDUH. 2023 NSDUH Detailed Tables | CBHSQ Data. 2023. https://www.samhsa.gov/data/report/2023-nsduh-detailed-tables

[39] Patrick ME, Kloska DD, Terry-McElrath YM, Lee CM, O’Malley PM, Johnston LD. Patterns of simultaneous and concurrent alcohol and marijuana use among adolescents. Am J Drug Alcohol Abuse. 2018;44(4):441–51. 10.1080/00952990.2017.1402335. 29261344 10.1080/00952990.2017.1402335 PMC6027645

[40] Patton JH, Stanford MS, Barratt ES. Factor structure of the barratt impulsiveness scale. J Clin Psychol. 1995;51(6):768–74. https://doi.org/10.1002/1097-4679(199511)51:6<768::AID-JCLP2270510607>3.0.CO;2-1 8778124 10.1002/1097-4679(199511)51:6<768::aid-jclp2270510607>3.0.co;2-1

[41] Pince CL, Stallsmith VT, Piercey CJ, Weldon K, Ruehrmund J, Dooley G, et al. Cannabis administration is associated with reduced alcohol consumption: evidence from a novel laboratory co-administration paradigm. Drug Alcohol Depend. 2025;112860. 10.1016/j.drugalcdep.2025.112860. 40915022 10.1016/j.drugalcdep.2025.112860 PMC13007497

[42] R Core Team. R: A language and environment for statistical computing [computer software]. R Foundation for Statistical Computing. 2025. https://www.R-project.org/

[43] Rothstein MC, Stamates AL. Impulsivity facets, social norms, and co-use of alcohol and cannabis. J Drug Issues. 2025. 10.1177/00220426251330821. 40895600 10.1177/00220426251330821 PMC12392365

[44] Saunders JB, Aasland OG, Babor TF, de la Fuente JR, Grant M. Development of the alcohol use disorders identification test (AUDIT): WHO collaborative project on early detection of persons with harmful alcohol consumption–II. Addiction. 1993;88(6):791–804. 10.1111/j.1360-0443.1993.tb02093.x. 8329970 10.1111/j.1360-0443.1993.tb02093.x

[45] Schacht JP, Voronin KE, Randall PK, Anton RF. Dopaminergic genetic variation influences aripiprazole effects on alcohol self-administration and the neural response to alcohol cues in a randomized trial. Neuropsychopharmacology. 2018;43(6):1247–56. 10.1038/npp.2017.298. 29362512 10.1038/npp.2017.298 PMC5916367

[46] Schacht JP, Im Y, Hoffman M, Voronin KE, Book SW, Anton RF. Effects of pharmacological and genetic regulation of COMT activity in alcohol use disorder: a randomized, placebo-controlled trial of tolcapone. Neuropsychopharmacology. 2022;47(11):1953–60. 10.1038/s41386-022-01335-z. 35523943 10.1038/s41386-022-01335-z PMC9073504

[47] Sokolovsky AW, Gunn RL, Micalizzi L, White HR, Jackson KM. Alcohol and marijuana co-use: consequences, subjective intoxication, and the operationalization of simultaneous use. Drug Alcohol Depend. 2020;212:107986. 10.1016/j.drugalcdep.2020.107986. 32417362 10.1016/j.drugalcdep.2020.107986 PMC7370922

[48] SPINELLA M. Normative data and a short form of the Barratt Impulsiveness Scale. Int J Neurosci. 2007;117(3):359–68. 10.1080/00207450600588881. 17365120 10.1080/00207450600588881

[49] Stamates AL, Linden-Carmichael AN, Miller SE, Feldstein Ewing SW. Impulsivity typologies and simultaneous alcohol and cannabis use. Exp Clin Psychopharmacol. 2023;31(3):599–604. 10.1037/pha0000608. 36174142 10.1037/pha0000608 PMC10107701

[50] Stein ER, Votaw VR, Swan JE, Witkiewitz K. Validity and measurement invariance of the addictions neuroclinical assessment incentive salience domain among treatment-seekers with alcohol use disorder. J Subst Abuse Treat. 2021;122:108227. 10.1016/j.jsat.2020.108227. 33509416 10.1016/j.jsat.2020.108227 PMC7846818

[51] Stellern J, Xiao KB, Grennell E, Sanches M, Gowin JL, Sloan ME. Emotion regulation in substance use disorders: a systematic review and meta-analysis. Addiction. 2023;118(1):30–47. 10.1111/add.16001. 35851975 10.1111/add.16001 PMC10087816

[52] Stephens RS, Roffman RA, Curtin L. Comparison of extended versus brief treatments for marijuana use. J Consult Clin Psychol. 2000;68(5):898–908. 10.1037/0022-006X.68.5.898. 11068976

[53] Stevens AK, Gunn RL, Boyle HK, White HR, Jackson KM. Unplanned versus planned simultaneous alcohol and cannabis use in the daily lives of a predominantly white college student sample: what are the motives, contexts, and outcomes? Psychol Addict Behav. 2022;36(3):243–53. 10.1037/adb0000813. 35113586 10.1037/adb0000813 PMC9106840

[54] Stevenson BL, Dvorak RD, Kramer MP, Peterson RS, Dunn ME, Leary AV, et al. Within- and between-person associations from mood to alcohol consequences: the mediating role of enhancement and coping drinking motives. J Abnorm Psychol. 2019;128(8):813–22. 10.1037/abn0000472. 31657596 10.1037/abn0000472

[55] Subbaraman MS, Kerr WC. Simultaneous versus concurrent use of alcohol and cannabis in the National Alcohol Survey. Alcohol Clin Exp Res. 2015;39(5):872–9. 10.1111/acer.12698. 25872596 10.1111/acer.12698 PMC4399000

[56] Thompson K, Holley M, Sturgess C, Leadbeater B. Co-use of alcohol and cannabis: longitudinal associations with mental health outcomes in young adulthood. Int J Environ Res Public Health. 2021;18(7):3652. 10.3390/ijerph18073652. 33807491 10.3390/ijerph18073652 PMC8037602

[57] Tomko RL, Bountress KE, Gray KM. Personalizing substance use treatment based on pre-treatment impulsivity and sensation seeking: a review. Drug Alcohol Depend. 2016;167:1–7. 10.1016/j.drugalcdep.2016.07.022. 27515725 10.1016/j.drugalcdep.2016.07.022 PMC5037032

[58] Tucker JS, Seelam R, Green HD, Rodriguez A, Pollard MS. Alcohol and cannabis co-use in a national sample of U.S. adults ages 30–80. Addict Behav. 2023;142:107663. 10.1016/j.addbeh.2023.107663. 36842190 10.1016/j.addbeh.2023.107663 PMC10049786

[59] VanderVeen JD, Plawecki MH, Millward JB, Hays J, Kareken DA, O’Connor S, et al. Negative urgency, mood induction, and alcohol seeking behaviors. Drug Alcohol Depend. 2016;165:151–8. 10.1016/j.drugalcdep.2016.05.026. 27291583 10.1016/j.drugalcdep.2016.05.026 PMC5045899

[60] Vieira JL, Coelho SG, Snaychuk LA, Tabri N, Dawson SJ, Hodgins DC, et al. Mental health and dispositional predictors of simultaneous versus concurrent cannabis and alcohol use in a Canadian context. Cannabis. 2024;7(3):41–60. 10.26828/cannabis/2024/000256. 39781556 10.26828/cannabis/2024/000256 PMC11705038

[61] Votaw VR, Pearson MR, Stein E, Witkiewitz K. The addictions neuroclinical assessment negative emotionality domain among treatment-seekers with alcohol use disorder: construct validity and measurement invariance. Alcohol Clin Exp Res. 2020;44(3):679–88. 10.1111/acer.14283. 31957027 10.1111/acer.14283 PMC7069798

[62] Waddell JT. Between- and within-group effects of alcohol and cannabis co-use on AUD/CUD in the NSDUH 2002–2019. Drug Alcohol Depend. 2021;225:108768. 10.1016/j.drugalcdep.2021.108768. 34049100 10.1016/j.drugalcdep.2021.108768

[63] Waddell JT. Hierarchical and mediated relations between internalizing symptoms, alcohol and cannabis co-use, and AUD. Addict Res Theory. 2022;30(3):213–9. 10.1080/16066359.2021.1999936.

[64] Waddell JT, Blake AJ, Chassin L. Relations between impulsive personality traits, alcohol and cannabis co-use, and negative alcohol consequences: a test of cognitive and behavioral mediators. Drug Alcohol Depend. 2021;225:108780. 10.1016/j.drugalcdep.2021.108780. 34049097 10.1016/j.drugalcdep.2021.108780 PMC9258026

[65] Waddell JT, Corbin WR, Grimm KJ, Metrik J, Lee CM, Trull TJ. Within-episode relations among simultaneous alcohol and cannabis use and continued drinking: the role of momentary subjective responses, craving, and drinking context. Alcohol Clin Exp Res. 2024;48(11):2175–87. 10.1111/acer.15451. 10.1111/acer.15451 PMC11913312 39367536

[66] Waddell JT, Gunn RL, Corbin WR, Borsari B, Metrik J. Drinking less on cannabis use days: the moderating role of UPPS-P impulsive personality traits. Psychol Addict Behav. 2021;35(6):737–48. 10.1037/adb0000727. 34591516 10.1037/adb0000727 PMC8484778

[67] Waddell JT, Howe LK. Relations among adolescent alcohol and cannabis co-use, adolescent impulsive traits, and prospective change in impulsive traits into emerging adulthood. Cannabis. 2023;6(2):89. 37484051 10.26828/cannabis/2023/000162 PMC10361807

[68] Waddell JT, Jager J, Chassin L. Maturing out of alcohol and cannabis co-use: a test of patterns and personality predictors. Alcohol Clin Exp Res. 2022;46(8):1603–15. 10.1111/acer.14898. 35994040 10.1111/acer.14898 PMC10325930

[69] Wardell JD, Farrelly KN, Moore A, Fox N, Coelho SG, Cunningham JA, et al. Internalizing symptoms are indirectly associated with simultaneous alcohol and cannabis use through specific motives for simultaneous use: a longitudinal study of young adults. Alcohol Clin Exp Res. 2025;49(10):2319–33. 10.1111/acer.70147. 10.1111/acer.70147 PMC12519063 40923811

[70] Whiteside SP, Lynam DR, Miller JD, Reynolds SK. Validation of the UPPS impulsive behaviour scale: a four-factor model of impulsivity. Eur J Pers. 2005;19(7):559–74. 10.1002/per.556.

[71] Wilcox CE, Pommy JM, Adinoff B. Neural circuitry of impaired emotion regulation in substance use disorders. Am J Psychiatry. 2016. 10.1176/appi.ajp.2015.15060710. 26771738 10.1176/appi.ajp.2015.15060710 PMC4979988

[72] Wycoff AM, Trull TJ. Affective reinforcement of simultaneous versus single use of alcohol and cannabis. Drug Alcohol Depend. 2025;270:112612. 10.1016/j.drugalcdep.2025.112612. 40020640 10.1016/j.drugalcdep.2025.112612 PMC11951136

[73] Zuckerman M, Kuhlman DM, Joireman J, Teta P, Kraft M. A comparison of three structural models for personality: the big three, the big five, and the alternative five. J Personal Soc Psychol. 1993;65(4):757–68. 10.1037/0022-3514.65.4.757.

