# Supplementary material for: Behavioral phenotypes associated with cannabis and alcohol substitution
Source: Harm Reduct J. 2026 Mar 6;23:74. doi: 10.1186/s12954-026-01432-y (PMC13078031; doi:10.1186/s12954-026-01432-y)
Supplement: Supplementary file 1 — Supplementary Material 1 [file 12954_2026_1432_MOESM1_ESM.docx]

**Methods**

**Participant Eligibility Criteria**

1. Between the ages of 21–60
2. Heavy drinking for at least 3 months; defined as > 4 drinks on any day or more than 14 drinks per week for men; more than 3 drinks on any day or more than 7 drinks per week for women
3. Regular flower cannabis use, defined as use > = 3 times per week for the past 3 months
4. No daily tobacco use
5. No history of significant mental health disorders (e.g., bipolar disorder, schizophrenia);
6. No substance use disorder diagnosis and not currently seeking treatment for a substance use disorder
7. Not currently pregnant, breastfeeding or trying to become pregnant
8. No current use of psychotropic medications besides antidepressants
9. No illicit drug use in the past 60 days (verified by self-report and urine drug screen)
10. No major medical conditions that contraindicate the use of alcohol or cannabis
11. No current immune or GI disorder
12. No use of probiotics or antibiotics in the past 3 months.
13. Must be willing to abstain from cannabis use for 14 days during the study.

**Behavioral Phenotyping Measures**

Group descriptive statistics and results from Welch’s ANOVAs (*F_w_*) or Kruskal-Wallis (𝜒^2^) tests are reported for each measure.

***Incentive Salience***

**Alcohol Use Disorders Identification Test (AUDIT)**

The AUDIT is an 11-item scale used to measure alcohol use disorder symptomatology (Saunders et al., 1993), which has demonstrated validity and reliability in a wide variety of samples (Meneses-Gaya et al., 2009). Scores range from 0-40, with the minimum threshold for clinical diagnosis of mild AUD being a score of 7, while scores above 16 represent the clinical cut-off for moderate to severe AUD (Ingesson-Hammarberg et al., 2024).

| *Alcohol Use Disorders Identification Test (AUDIT)*  𝜒^2^(2) = 1.27, *p* = .53 | | | | |
| --- | --- | --- | --- | --- |
|  | *M* | *SD* | *Mdn* | *IQR* |
| Substituters | 9.73 | 3.52 | 9.00 | 4.00 |
| Non-substituters | 12.56 | 6.68 | 10.50 | 8.00 |
| Complementers | 10.30 | 5.18 | 9.00 | 6.50 |

**Penn Alcohol Craving Scale (PACS)**

The PACS is a 5-item scale (score range 0-30) used to measure alcohol craving over the past week, which has demonstrated validity and reliability (Flannery et al., 1999). PACS scores have also been shown to be strong predictors of drinking or relapse in AUD treatment (Flannery et al., 2003).

| *Penn Alcohol Craving Scale (PACS)*  𝜒^2^(2) = 0.97, *p* = .62 | | | | |
| --- | --- | --- | --- | --- |
|  | *M* | *SD* | *Mdn* | *IQR* |
| Substituters | 7.74 | 4.92 | 6.00 | 5.50 |
| Non-substituters | 10.75 | 5.93 | 9.50 | 9.75 |
| Complementers | 9.42 | 5.79 | 9.00 | 9.00 |

**Marijuana Dependence Scale (MDS)**

The MDS is an 11-item questionnaire that assesses symptoms of cannabis dependence using true/false statements, based on DSM-IV TR criteria, where endorsement of three or more items indicates cannabis dependence (Stephens et al., 2000). The MDS has previously been shown to have adequate reliability (Stephens et al., 2000).

| *Marijuana Dependence Scale (MDS)*  *F_w_*(15.2,2) = 0.97, *p* = .40 | | | | |
| --- | --- | --- | --- | --- |
|  | *M* | *SD* | *Mdn* | *IQR* |
| Substituters | 2.91 | 2.72 | 2.00 | 3.50 |
| Non-substituters | 2.00 | 1.71 | 2.00 | 4.00 |
| Complementers | 3.14 | 3.33 | 2.00 | 4.50 |

**Marijuana Craving Questionnaire**

The MCQ is a 47-item questionnaire that captures cannabis craving in the moment using a 7-point Likert scale (Strongly Disagree = 1 and Strongly Agree = 7), with a general factor and four subscales pertaining to different dimensions of craving: compulsivity, emotionality, expectancy, and purposefulness, which are scored based on average responses to constituent items (Heishman et al., 1997). The MCQ has demonstrated reliability and validity (Singleton et al., 2002), in addition to predicting cannabis use amongst individuals trying to cut down (Enkema et al., 2020).

| *Marijuana Craving Questionnaire General Factor*  *F_w_*(21.5,2) = 0.53, *p* = .60 | | | | |
| --- | --- | --- | --- | --- |
|  | *M* | *SD* | *Mdn* | *IQR* |
| Substituters | 5.36 | 1.03 | 5.41 | 1.83 |
| Non-substituters | 5.22 | 0.73 | 5.15 | 0.49 |
| Complementers | 5.50 | 0.53 | 5.41 | 0.62 |
| *Compulsivity Subscale*  *F_w_*(21.3,2) = 1.3, *p* = .30 | | | | |
|  | *M* | *SD* | *Mdn* | *IQR* |
| Substituters | 5.44 | 1.11 | 5.14 | 1.30 |
| Non-substituters | 8.87 | 4.94 | 0.78 | 5.00 |
| Complementers | 5.12 | 0.58 | 5.00 | 0.57 |
| *Emotionality Subscale*  *F_w_*(21.4,2) = 2.41, *p* = .11 | | | | |
|  | *M* | *SD* | *Mdn* | *IQR* |
| Substituters | 5.34 | 2.50 | 6.25 | 4.00 |
| Non-substituters | 6.36 | 1.67 | 6.87 | 2.88 |
| Complementers | 6.89 | 1.26 | 7.25 | 2.00 |
| *Expectancy Subscale*  *F_w_*(17.8,2) = 2.87, *p* = .08 | | | | |
|  | *M* | *SD* | *Mdn* | *IQR* |
| Substituters | 5.27 | 1.21 | 5.00 | 1.67 |
| Non-substituters | 4.40 | 1.04 | 4.33 | 1.17 |
| Complementers | 4.95 | 0.99 | 4.67 | 1.33 |
| *Purposefulness Subscale*  *F_w_*(17.1,2) = .10, *p* = .9 | | | | |
|  | *M* | *SD* | *Mdn* | *IQR* |
| Substituters | 5.30 | 1.24 | 7.00 | 1.44 |
| Non-substituters | 5.17 | 1.30 | 5.33 | 1.75 |
| Complementers | 5.10 | 1.20 | 5.00 | 1.67 |

***Negative Emotionality***

**Beck Depression Inventory II (BDI-II)**

The BDI-II is a 21-item scale utilized to assess symptoms of depression over the past 2 weeks, including three subscales capturing cognitive, affective, and somatic symptoms (Beck et al., 1996; Buckley et al., 2001), which has shown reliability and validity (Wang & Gorenstein, 2013). Higher scores indicate more severe depression symptoms (0-63, > 29 severe) (Beck et al., 1996).

| *Beck Depression Inventory II*  𝜒^2^(2) = 8.79, *p* = .01 | | | | |
| --- | --- | --- | --- | --- |
|  | *M* | *SD* | *Mdn* | *IQR* |
| Substituters | 6.00 | 5.53 | 5.00 | 10.00 |
| Non-substituters | 12.31 | 8.62 | 11.50 | 14.25 |
| Complementers | 3.14 | 2.34 | 3.00 | 3.50 |

**Beck Anxiety Inventory (BAI)**

The BAI is a 21-item scale (score range 0-63) assessing physical and cognitive anxiety symptoms over the past 2 weeks, with greater scores indicating greater severity (0-63, > 26 severe) (Beck et al., 1988). The BAI has demonstrated reliability and validity (Fydrich et al., 1992)

| *Beck Anxiety Inventory*  𝜒^2^(2) = 2.94, *p* = .23 | | | | |
| --- | --- | --- | --- | --- |
|  | *M* | *SD* | *Mdn* | *IQR* |
| Substituters | 3.35 | 3.41 | 2.00 | 5.00 |
| Non-substituters | 5.69 | 5.66 | 4.50 | 3.00 |
| Complementers | 3.57 | 2.93 | 5.00 | 4.00 |

**Depression Anxiety Stress Scale**

The DASS is a 42-item scale with 3 subscales (score range 0-42) that capture symptoms of depression, anxiety, and tension/stress (Lovibond & Lovibond, 1995), which has demonstrated reliability and validity (Coker et al., 2018). Unlike the BDI, the DASS does not capture somatic symptoms of depression and clinical cutoffs vary by subscale (Lovibond & Lovibond, 1995).

| *DASS Depression*  𝜒^2^(2) = 6.60, *p* = .04 | | | | |
| --- | --- | --- | --- | --- |
|  | *M* | *SD* | *Mdn* | *IQR* |
| Substituters | 2.30 | 3.29 | 1.00 | 2.50 |
| Non-substituters | 5.94 | 6.03 | 5.00 | 6.25 |
| Complementers | 2.71 | 1.89 | 2.00 | 1.50 |
| *DASS Anxiety*  𝜒^2^(2) = 0.40, *p* = .82 | | | | |
|  | *M* | *SD* | *Mdn* | *IQR* |
| Substituters | 3.61 | 3.43 | 3.00 | 5.00 |
| Non-substituters | 4.13 | 3.51 | 3.00 | 9.50 |
| Complementers | 3.28 | 2.93 | 3.00 | 2.50 |
| *DASS Stress*  𝜒^2^(2) = 2.03, *p* = .36 | | | | |
|  | *M* | *SD* | *Mdn* | *IQR* |
| Substituters | 5.65 | 3.76 | 5.00 | 5.00 |
| Non-substituters | 8.19 | 5.56 | 9.00 | 2.25 |
| Complementers | 5.71 | 3.20 | 6.00 | 5.00 |

***Executive Function***

**Urgency, Premeditation, Perseverance, Sensation Seeking, and Positive urgency Impulsive Behavior Scale (UPPS-P)**

The short-form UPPS-P is a 20-item version of the original 59-item scale, comprised of 5 subscales capturing different dimensions of trait impulsivity, including negative/positive urgency, lack of premeditation/perseverance, and sensation seeking (Cyders et al., 2014; Whiteside et al., 2005). Subscale scores represent averages of constituent items (1-4 Likert scale) (Cyders et al., 2014; Whiteside et al., 2005). The short-form UPPS-P has demonstrated validity and reliability (Cyders et al., 2014).

| *Positive Urgency Subscale*  𝜒^2^(2) = 4.23, *p* = .12 | | | | |
| --- | --- | --- | --- | --- |
|  | *M* | *SD* | *Mdn* | *IQR* |
| Substituters | 1.62 | 0.66 | 1.50 | 0.75 |
| Non-substituters | 1.95 | 0.56 | 2.00 | 0.31 |
| Complementers | 1.57 | 0.55 | 1.50 | 0.75 |
| *Negative Urgency Subscale*  𝜒^2^(2) = 5.32, *p* = .07 | | | | |
|  | *M* | *SD* | *Mdn* | *IQR* |
| Substituters | 1.71 | 0.58 | 1.75 | 0.75 |
| Non-substituters | 2.08 | 0.39 | 2.00 | 0.38 |
| Complementers | 1.93 | 0.66 | 2.25 | 1.13 |
| *Lack of Premeditation Subscale*  𝜒^2^(2) = 6.06, *p* = .05 | | | | |
|  | *M* | *SD* | *Mdn* | *IQR* |
| Substituters | 1.50 | 0.56 | 1.25 | 0.88 |
| Non-substituters | 1.81 | 0.36 | 1.75 | 0.50 |
| Complementers | 1.54 | 0.27 | 1.50 | 0.25 |
| *Lack of Perseverance Subscale*  𝜒^2^(2) = 0.95, *p* = .62 | | | | |
|  | *M* | *SD* | *Mdn* | *IQR* |
| Substituters | 1.72 | 0.40 | 1.75 | 0.50 |
| Non-substituters | 1.59 | 0.35 | 1.63 | 0.31 |
| Complementers | 1.75 | 0.63 | 1.75 | 0.88 |
| *Sensation Seeking Subscale*  𝜒^2^(2) = 0.14, *p* = .93 | | | | |
|  | *M* | *SD* | *Mdn* | *IQR* |
| Substituters | 3.00 | 0.66 | 3.25 | 1.00 |
| Non-substituters | 2.97 | 0.96 | 3.37 | 1.63 |
| Complementers | 3.04 | 0.59 | 3.00 | 0.88 |

**Barratt Impulsiveness Scale (BIS-15) short-form**

The short-form BIS is a 15-item version of the original 30-item scale which measures three dimensions of trait impulsivity (score range 15-60): non-planning, motor, and attentional (Patton et al., 1995; Spinella, 2007). The BIS-15 has demonstrated reliability and validity (Meule et al., 2015).

| *Barratt Impulsiveness Scale Total*  𝜒^2^(2) = 1.60, *p* = .44 | | | | |
| --- | --- | --- | --- | --- |
|  | *M* | *SD* | *Mdn* | *IQR* |
| Substituters | 38.70 | 2.60 | 38.00 | 4.00 |
| Non-substituters | 38.30 | 1.94 | 38.00 | 3.00 |
| Complementers | 37.42 | 1.13 | 38.00 | 1.50 |
| *Attentional Subscale*  𝜒^2^(2) = 0.87, *p* = .65 | | | | |
|  | *M* | *SD* | *Mdn* | *IQR* |
| Substituters | 12.00 | 1.78 | 12.00 | 1.50 |
| Non-substituters | 12.53 | 1.06 | 13.00 | 1.00 |
| Complementers | 11.43 | 2.07 | 12.00 | 3.50 |
| *Motor Subscale*  𝜒^2^(2) = 0.12, *p* = .94 | | | | |
|  | *M* | *SD* | *Mdn* | *IQR* |
| Substituters | 12.61 | 1.85 | 12.00 | 2.00 |
| Non-substituters | 12.25 | 1.61 | 12.00 | 3.00 |
| Complementers | 12.71 | 1.11 | 13.00 | 1.50 |
| *Non-planning Subscale*  𝜒^2^(2) = 0.84, *p* = .94 | | | | |
|  | *M* | *SD* | *Mdn* | *IQR* |
| Substituters | 14.10 | 2.70 | 14.00 | 3.00 |
| Non-substituters | 13.50 | 1.97 | 14.00 | 3.00 |
| Complementers | 13.30 | 1.40 | 14.00 | 1.50 |

**Impaired Control Scale (ICS)**

The ICS is a 25-item scale (score range 0-100) employed to measure difficulties controlling drinking, including subscales on perceived (0-40), attempted (0-20), and failed control (0-40) (Heather et al., 1993), which has demonstrated validity and internal consistency in samples of both social and treatment-engaged drinkers (Marsh et al., 2002).

| *Impaired Control Scale (ICS) Total*  𝜒^2^(2) = 0.29, *p* = .87 | | | | |
| --- | --- | --- | --- | --- |
|  | *M* | *SD* | *Mdn* | *IQR* |
| Substituters | 46.82 | 7.40 | 48.00 | 8.75 |
| Non-substituters | 48.67 | 7.41 | 47.50 | 11.75 |
| Complementers | 48.28 | 12.98 | 45.00 | 9.50 |
| *Perceived Control Subscale*  𝜒^2^(2) = 0.42, *p* = .81 | | | | |
|  | *M* | *SD* | *Mdn* | *IQR* |
| Substituters | 7.44 | 5.75 | 8.00 | 11.00 |
| Non-substituters | 8.87 | 4.48 | 8.50 | 6.25 |
| Complementers | 6.57 | 5.22 | 8.00 | 6.50 |
| *Attempted Control Subscale*  𝜒^2^(2) = 0.74, *p* = .69 | | | | |
|  | *M* | *SD* | *Mdn* | *IQR* |
| Substituters | 20.21 | 3.77 | 21.00 | 5.50 |
| Non-substituters | 19.62 | 3.68 | 19.00 | 5.50 |
| Complementers | 19.57 | 2.82 | 20.00 | 2.00 |
| *Failed Control Subscale*  𝜒^2^(2) = 0.43, *p* = .81 | | | | |
|  | *M* | *SD* | *Mdn* | *IQR* |
| Substituters | 19.10 | 4.80 | 18.50 | 6.50 |
| Non-substituters | 20.18 | 7.21 | 19.00 | 6.00 |
| Complementers | 22.14 | 12.43 | 19.00 | 3.50 |

**Impulsive Sensation Seeking Scale (ImpSS)**

The ImpSS is a 19-item scale with true/false statements (score range 0-19) that measures aspects of personality and impulsivity related to sensation seeking (Zuckerman et al., 1993), which has shown to have good reliability and validity (McDaniel & Mahan, 2008).

| *Impulsive Sensation Seeking Scale (ImpSS) Total*  *F_w_*(18.6,2) = 0.84, *p* = .45 | | | | |
| --- | --- | --- | --- | --- |
|  | *M* | *SD* | *Mdn* | *IQR* |
| Substituters | 7.17 | 4.40 | 7.00 | 4.00 |
| Non-substituters | 7.56 | 3.69 | 6.50 | 5.25 |
| Complementers | 9.14 | 3.24 | 8.00 | 3.00 |

**Difficulties in Emotion Regulation Scale (DERS)**

The DERS-18 is a shortened form of the DERS-32 that measures challenges across different dimensions of emotion regulation (score range 18-90), which shows good reliability and validity (Gratz & Roemer, 2004; Victor & Klonsky, 2016). The DERS-18 includes six subscales, which reflect Lack of Emotional Awareness, Lack of Emotional Clarity, Difficulties Engaging in Goal-Directed Behavior, Impulse Control Difficulties, Non-acceptance of Emotional Responses, and Limited Access to Emotion Regulation Strategies (subscale score range 3-15) (Gratz & Roemer, 2004; Victor & Klonsky, 2016).

| *Difficulties in Emotion Regulation Scale Total*  𝜒^2^(2) = 2.51, *p* = .29 | | | | |
| --- | --- | --- | --- | --- |
|  | *M* | *SD* | *Mdn* | *IQR* |
| Substituters | 33.65 | 10.67 | 31.00 | 14.50 |
| Non-substituters | 37.18 | 8.45 | 34.00 | 11.75 |
| Complementers | 31.86 | 7.63 | 33.00 | 11.00 |
| *Emotional Non-acceptance*  𝜒^2^(2) = 3.34, *p* = .19 | | | | |
|  | *M* | *SD* | *Mdn* | *IQR* |
| Substituters | 6.39 | 2.57 | 6.00 | 3.00 |
| Non-substituters | 7.00 | 1.67 | 7.00 | 2.25 |
| Complementers | 5.57 | 2.15 | 5.00 | 3.00 |
| *Difficulties in Goal-directed Behavior*  𝜒^2^(2) = 1.63, *p* = .44 | | | | |
|  | *M* | *SD* | *Mdn* | *IQR* |
| Substituters | 6.30 | 2.70 | 6.00 | 4.50 |
| Non-substituters | 6.53 | 2.53 | 5.50 | 4.25 |
| Complementers | 5.43 | 2.44 | 4.00 | 4.50 |
| *Impulse Control Difficulties*  𝜒^2^(2) = 1.27, *p* = .53 | | | | |
|  | *M* | *SD* | *Mdn* | *IQR* |
| Substituters | 5.13 | 2.36 | 5.00 | 3.00 |
| Non-substituters | 5.50 | 1.67 | 5.00 | 3.00 |
| Complementers | 4.87 | 2.27 | 4.00 | 3.50 |
| *Lack of Emotional Awareness*  𝜒^2^(2) = 3.10, *p* = .21 | | | | |
|  | *M* | *SD* | *Mdn* | *IQR* |
| Substituters | 5.30 | 1.87 | 5.00 | 2.50 |
| Non-substituters | 6.37 | 1.93 | 6.00 | 3.00 |
| Complementers | 5.71 | 2.70 | 5.00 | 2.50 |
| *Limited Access to Emotion Regulation Strategies*  𝜒^2^(2) = 2.43, *p* = .0 | | | | |
|  | *M* | *SD* | *Mdn* | *IQR* |
| Substituters | 5.48 | 2.23 | 5.00 | 2.00 |
| Non-substituters | 5.19 | 1.72 | 5.00 | 1.25 |
| Complementers | 4.14 | 0.90 | 4.00 | 1.50 |
| *Lack of Emotional Clarity*  𝜒^2^(2) = 5.55, *p* = .06 | | | | |
|  | *M* | *SD* | *Mdn* | *IQR* |
| Substituters | 5.10 | 2.13 | 5.00 | 2.50 |
| Non-substituters | 6.56 | 2.30 | 6.00 | 1.48 |
| Complementers | 6.14 | 2.54 | 6.00 | 3.00 |

**Cannabinoid Analysis by LC−MS/MS**

All cannabinoid standards and deuterium-labeled internal standards were purchased from Cerilliant (Round Rock, TX, USA). Water, methanol, and acetonitrile (LC–MS grade) were purchased from Millipore (Burlington, MA, USA). Formic acid (LC−MS grade) was obtained from Sigma-Aldrich (St. Louis, MO, USA). Captiva EMR-Lipid columns (1 mL, 40 mg) were purchased from Agilent Technologies (Santa Clara, CA, USA). Liquid chromatography columns were purchased from Restek Inc. (Bellefonte, PA, USA).

Matrix-matched calibrators and controls were prepared by the addition of appropriate volumes of methanolic stock standard mixtures (0.01, 0.1,1.0, or 10 µg/mL of each cannabinoid) to 100 µL of cannabinoid-free plasma to produce calibrators at 0.5, 1, 5, 10, 50, 100, 500, and 1000ng/mL. Quality control samples were prepared at 5 ng/mL, 750, and 700 ng/ml for each analyte. Quality control samples were run after every 20 subject samples, with an expected accuracy of +/- 20%.

Subject plasma samples, matrix-matched standards, and quality control samples were prepared for LC−MS/MS analysis by protein precipitation and lipid removal. Ten microliters of the internal standard solution (0.3 µg/mL for ∆^9^-THC-d_3_, 0.1 µg/mL 11-hydroxy-∆^9^-THC-d_3_, and 0.8 µg/mL 11-carboxy-∆^9^-THC-d_9_) was added to 100 μL of plasma sample and vortexed in a polypropylene microcentrifuge tube. 600 μL of ice-cold acetonitrile/methanol (85%/15%) was added dropwise while vortexing to precipitate proteins. Samples were centrifuged at 14,000 rpms and supernatants were transferred to Captiva EMR-Lipid columns for lipid removal. Using a positive-pressure manifold, 3 psi of pressure was applied to elute the samples through the columns. Eluents were collected into clean glass test tubes and dried under nitrogen at 45°C. Eluents were reconstituted in 100 µL of water/methanol (50%/50%) with 0.1% formic acid and transferred to autosampler vials with pulled-point inserts for LC−MS/MS analysis.

Samples were analyzed with an Agilent 1290 Infinity II liquid chromatograpy coupled to an Agilent 6475 triple quadruple mass spectrometer equipped with an Agilent Jet Stream electrospray ionization source (Agilent, Santa Clara, CA). Cannabinoids were chromatographically separated on a Restek Raptor biphenyl column (3.0 × 50 mm, 2.7 μm) and held at 40°C. A sample volume of 10 μL was injected, and a mixture of water with 0.1% formic acid (A) and methanol with 0.1% formic acid (B) was introduced at a flow rate of 0.4 mL/min. Gradient elution started at 40% B, which was increased to 70% B over 1 min and subsequently to 75% B over 3.5 min, and ended at 100% B at 6 min. The ionization source conditions used were as follows: positive polarity, nebulizer pressure of 45 psi; gas flow of 12 L/min at 300°C; sheath gas flow of 12 L/min at 375°C; capillary voltage of 3500 V; and nozzle voltage of 2000V. The ion transitions monitored are displayed in Table 1. Analytes were confirmed by the retention time and the product ion ratio (± 20%) correlation between the sample peaks and corresponding standards. Data collection and processing were performed by using Agilent MassHunter quantitative software (v.B.12.01). Quantitation was performed with linear regression using 8-point calibration curves from 0.5 ng/mL to 1000 ng/mL for each analyte and the limit of detection was 0.25 ng/ml for each analyte.

Any values that were not detected (ND) were replaced with zeros. The limit of quantitation was 0.5 ng/ml for each analyte. Anything below the limit of quantitation was converted to .25 ng/ml (LOQ/2).

Table 1. LC−MS/MS ion transitions monitored for cannabinoids in human plasma.

| **Analyte Name** | **Precursor Ion** | **Product Ion** | **Fragmentor (V)** | **Collision Energy (V)** | **Polarity** |
| --- | --- | --- | --- | --- | --- |
| 11-carboxy-∆^9^-THC | 345.2 | 299.2 | 130 | 16 | Positive |
| 11-carboxy-∆^9^-THC | 345.2 | 119 | 130 | 32 | Positive |
| 11-carboxy-∆^9^-THC-d_9_ | 354.3 | 336.2 | 130 | 12 | Positive |
| 11-carboxy-∆^9^-THC-d_9_ | 354.3 | 308.2 | 130 | 16 | Positive |
| 11-hydroxy-∆^9^-THC | 331.2 | 313.2 | 112 | 9 | Positive |
| 11-hydroxy-∆^9^-THC | 331.2 | 193.1 | 112 | 21 | Positive |
| 11-hydroxy-∆^9^-THC-d_3_ | 334.2 | 316.2 | 112 | 12 | Positive |
| 11-hydroxy-∆^9^-THC-d_3_ | 334.3 | 105.1 | 112 | 48 | Positive |
| ∆^9^-THC | 315.2 | 193.1 | 122 | 20 | Positive |
| ∆^9^-THC | 315.2 | 123 | 122 | 32 | Positive |
| ∆^9^-THC-d_3_ | 318.2 | 196.1 | 122 | 20 | Positive |
| ∆^9^-THC-d_3_ | 318.2 | 123 | 122 | 32 | Positive |
